# The Cardiovascular Protective Function of Natural Compounds Through AMPK/SIRT1/PGC‐1α Signaling Pathway

**DOI:** 10.1002/fsn3.4553

**Published:** 2024-11-08

**Authors:** Sohrab Rahmani, Ali Roohbakhsh, Vahid Pourbarkhordar, Gholamreza Karimi

**Affiliations:** ^1^ Student Research Committee Mashhad University of Medical Sciences Mashhad Iran; ^2^ Department of Pharmacodynamics and Toxicology, School of Pharmacy Mashhad University of Medical Sciences Mashhad Iran; ^3^ Pharmaceutical Research Center, Institute of Pharmaceutical Technology Mashhad University of Medical Sciences Mashhad Iran

**Keywords:** AMPK, heart, mitochondria, natural compounds, SIRT1/PGC‐1α pathway

## Abstract

Cardiovascular disease (CVD) poses a major risk to human health and exert a heavy burden on individuals, society, and healthcare systems. Therefore, it is critical to identify CVD's underlying mechanism(s) and target them using effective agents. Natural compounds have shown promise as antioxidants with cardioprotective functions against CVD injuries due to their antioxidative solid capacity and high safety profile. Several CVDs, such as heart failure, ischemia/reperfusion, atherosclerosis, and cardiomyopathies, are closely linked to mitochondrial dysfunction. It is well established that activating the AMPK/SIRT1/PGC‐1α pathway during CVD promotes mitochondrial function. Therefore, targeting the AMPK/SIRT1/PGC‐1α pathway provides a foundation for novel therapeutic strategies to combat CVD. A key goal of our search was to find natural compounds that target this biological pathway and have beneficial effects on CVD.

## Graphical abstract

Cardiovascular disease (CVD) is a major threat to human health and imposes a heavy burden on individuals, society, and healthcare systems. A key goal of our search was to find natural compounds that target this biological pathway and have beneficial effects on CVD.

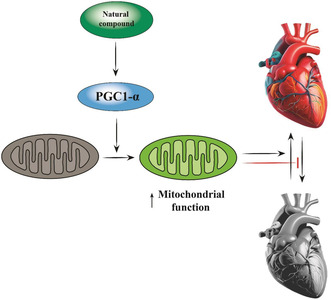

**Abbreviations**
AMPK: AMP‐activated protein kinase
ANT: adenine nucleotide translocator
ATP: adenosine triphosphate
Bak: Bcl‐2 antagonist killer Bcl‐2
Bax: Bcl‐2‐associated X protein
Bcl‐2: B‐cell lymphoma‐2: interacting protein 3
CVD: cardiovascular disease
CVeD: chronic venous disease
Cyt c: cytochrome c
DRP1: dynamin‐related protein 1
Fis1: fission protein 1
FoxO: Forkhead box‐O
FUNDC1: FUN14 domain containing 1
HF: heart failure
I/R: ischemia/reperfusion
IL‐6: interleukin‐6
IS: ischemic stroke
MAPK: mitogen‐activated protein kinase
MARCH: membrane‐associated RING‐CH
Mdivi‐1: mitochondrial division inhibitor 1
MEF: mitochondrial elongation factor
MFF: mitochondrial fission factor
MFN1: mitofusin‐1
MFN2: mitofusin‐2
MMP: mitochondrial membrane potential
MyD88: myeloid differentiation primary response 88
NLRP3: nucleotide‐binding domain and leucine‐rich repeat pyrin domain containing 3
NRF‐2: nuclear factor‐erythroid factor 2‐related factor 2
OMM: outer mitochondrial membrane
PGC‐1α: peroxisome proliferator‐activated receptor gamma coactivator 1‐alpha
PI3K: phosphatidylinositol 3‐Kinase
PINK1: PTEN‐induced putative kinase 1
PPARγ: peroxisome proliferator‐activated receptor γ
ROS: reactive oxygen species
SIRT1: sirtuin 1
TNF‐α: tumor necrosis factor‐α

## 1 Introduction

Cardiovascular disease (CVD) such as heart failure, cardiomyopathies, and coronary artery diseases are the major health concern affecting both men and women. It stands as one of the primary factors contributing to global mortality rates, causing at least one in four deaths each year (Molaei et al. 2023). CVD has the highest morbidity and mortality rates compared to other diseases, making it a severe threat to human health (Molaei et al. 2023). Mitochondria are the primary sites for reactive oxygen species (ROS) generation in the heart (Rahmani and Rezaei 2020). Given that the heart is a highly energy‐demanding organ, it requires a substantial amount of energy to function effectively; therefore, mitochondria are crucial organelles that produce energy in cardiomyocytes (Rahmani, Roohbakhsh, and Karimi 2023). Dysfunction of this organelle is an accompanying feature of many CVDs including myocardial ischemia, diabetic cardiomyopathy, heart failure, and cardiotoxicity (Rahmani, Roohbakhsh, and Karimi 2023).

Peroxisome proliferator–activated receptor gamma coactivator 1‐alpha (PGC‐1α), which was first identified in the late 1990s and belongs to the PGC‐1 family, plays a predominant role in regulating mitochondrial function in highly energy‐demanding organs like the heart, brain, liver, and skeletal muscles (Chambers and Wingert 2020; Helli et al. 2024). It is a transcriptional coactivator mainly found in tissues abundant in mitochondria. Coactivators are proteins that bind to transcription factors, such as NRF‐1, NRF‐2, ERRa, and mitochondrial transcription factor A (TFAM) that help to increase the expression of genes associated with oxidative phosphorylation (Chambers and Wingert 2020). The coactivators do not interact with DNA directly but amplify these genes' expression (Wu et al. 1999). PGC‐1α is a crucial regulator of oxidative metabolism that controls mitochondrial function and various cellular processes in different organs. For example, it regulates gluconeogenesis in the liver, thermogenesis in brown fat, and angiogenesis in skeletal muscle. PGC‐1α and PGC1‐β are proteins mostly found in mitochondria‐rich tissues, for example, the heart. However, studies have provided evidence that PRC only has a negligible effect on mitochondrial function. These proteins have a short lifespan because they are quickly marked with ubiquitin molecules and broken down by proteasomes (Trausch‐Azar et al. 2015).

The modification of PGC‐1α by posttranslational processes such as phosphorylation, acetylation, SUMOylation, and methylation is a topic of interest for cardiologists (Di et al. 2018; Riehle and Abel 2012). In particular, the deacetylation of PGC‐1α by sirtuins, particularly SIRT1, has been studied. Sirtuin proteins are distributed in various cellular compartments that coordinate the cellular response to stress through induction of mitochondrial oxidation and stress tolerance. In the nucleus, SIRT1 can promote accumulation of PGC‐1α which leads to gene transcription necessary for mitochondrial function and biogenesis (Zeng and Chen 2022). Phosphorylation of PGC‐1α can be regulated by upstream signaling proteins such as AMPK and MAPK, which improve its transcriptional activity (Rodgers et al. 2008). The activation of AMPK can regulate the function of mitochondria and the energy states of a cell by controlling its upstream silent information regulator 1 (SIRT1). Once SIRT1 is activated, it enhances the activity of PGC‐1. Therefore, the AMPK/SIRT1/PGC‐1α pathway plays a significant role in maintaining the function of mitochondria and biosynthesis (Rodgers et al. 2008).

In recent years, there has been a growing focus on researching the cardioprotective properties of natural compounds, attributed to their potential antioxidant, anti‐inflammatory, and antiapoptotic effects, that are believed to have fewer off‐target effects compared to chemical substances (Rahmani, Roohbakhsh, and Karimi 2023; Rahmani et al. 2023). Moreover, several studies have confirmed that natural compounds can activate the AMPK/SIRT1/PGC‐1α pathway and enhance mitochondrial functions to prevent pathological conditions (Li et al. 2022; Li, Cao, et al. 2021). Supporting this notion, many in vivo and in vitro cardiac disease models have confirmed this hypothesis (Li et al. 2022; Li, Cao, et al. 2021). Thus, the upregulation of the AMPK/SIRT1/PGC‐1α pathway could be an attractive target for treating pathological cardiovascular injuries like ischemia/reperfusion, hypertrophy, cardiotoxicities, and cardiomyopathies.

## 2 The Cardioprotective Effects of Natural Compounds Through AMPK/SIRT1/PGC‐1α Pathway

### 2.1 Myocardial Ischemia/Reperfusion

Myocardial ischemia is a condition in which normal blood flow gets restricted to the heart muscle, causing cardiac dysfunction, arrhythmias, myocardial infarction, and sudden death (Tibaut, Mekis, and Petrovic 2017). Ischemic heart disease serves as the foremost cause of mortality across the globe. Significant advancements have been made in preventing myocardial ischemia/reperfusion injury (MI/R) in the past few years (Tibaut, Mekis, and Petrovic 2017). Nevertheless, effectiveness remains limited. Recent studies have revealed that MI/R injuries result in mitochondrial abnormalities like increased oxidative damage, abrogated mitochondrial dynamics, and reduced biogenesis (Zhou et al. 2021). Strategies that maintain mitochondrial bioenergetics and biogenesis help the heart resist damage during stress (Zhou et al. 2021).

**FIGURE 1:**
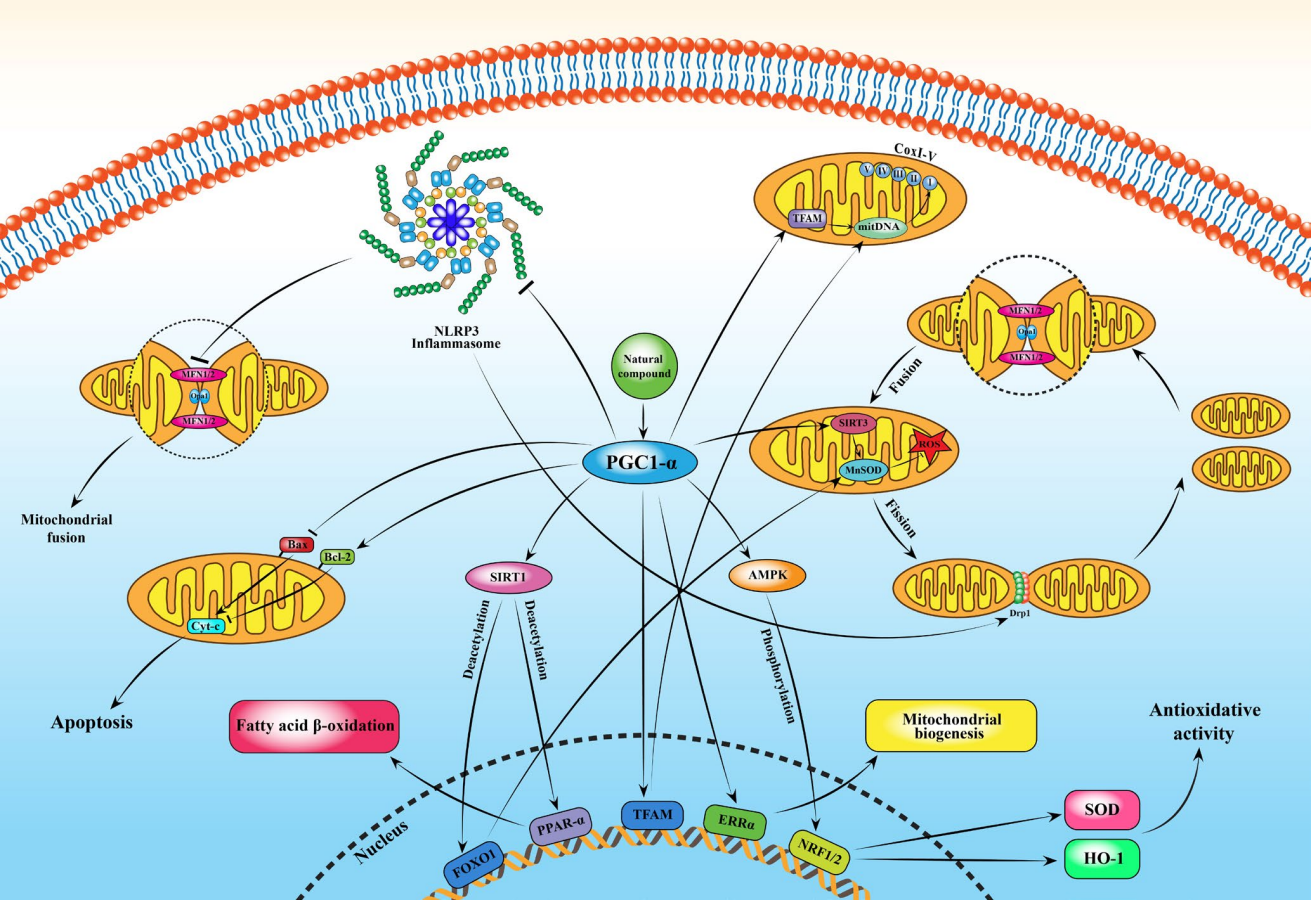
A schematic illustration of the natural compound–induced protective effects against CVD damage through AMPK/SIRT1/PGC‐1α signaling pathway. ↑ and → present the promote/activate, ⊥ and ↓ present the inhibitory/suppressive effects. AMPK, adenosine monophosphate–activated protein kinase; Drp1, dynamin‐related protein 1; HO‐1, heme oxygenase 1; MFN1, mitofusin‐1; MFN2, mitofusin‐2; NLRP3, nucleotide‐binding domain and leucine‐rich repeat pyrin domain containing 3; NRF‐2, NRF‐2 nuclear respiratory factor 1; PGC1α, peroxisome proliferator–activated receptor gamma coactivator 1‐alpha; TFAM, mitochondrial transcription factor A.

It has been demonstrated that the activation of AMPK reduces cardiac energy, which enhances mitochondrial energy production. Moreover, sirtuin 3 (SIRT3), which is vital in maintaining mitochondrial integrity and function, preserves myocardial mitochondrial function under oxidative stress conditions (Rato et al. 2014). Meanwhile, SIRT3 also functions as a crucial downstream effector of AMPK in regulating cardiomyocyte survival. It was demonstrated that naringenin, a flavanone derived from citrus fruits, reduces cardiac damage following ischemia/reperfusion injury by activating the AMPK/SIRT3 signaling pathway (Yu et al. 2019). By using AMPK1α siRNA, the cytoprotective effects of naringenin were blocked. Additionally, the knocking down of AMPK affected PGC‐1α and SIRT3 levels. The results indicate that mitochondrial dysfunction occurred during I/R, likely due to the downregulation of PGC‐1α and its downstream targets, TFAM and NRF‐1 (Yu et al. 2019). TFAM initiates the nuclear genes that encode subunits of the mitochondrial OXPHOS complex by binding to the mitochondrial promoters. NRF‐1 controls TFAM as a downstream target, specifically initiating the nuclear genes for the mitochondrial OXPHOS complex. A similar study conducted by Yu et al. (2017) showed that under diabetic conditions, melatonin treatment reduced mitochondrial oxidative stress and increased ATP production, as well as NRF‐1 and TFAM expressions in a diabetic MI/R rat model (Table 1). The melatonin‐treated group demonstrated markedly enhanced mitochondrial OXPHOS complex subunit expressions. Moreover, SIRT3 siRNA inhibited the upregulation of superoxide dismutase **(**SOD), NRF‐1, and TFAM induced by melatonin treatment without affecting p‐AMPK and PGC‐1α protein levels (Yu et al. 2017) (Table 1). Tilianin is a flavonoid glycoside found in various medicinal plants and is most commonly derived from *Dracocephalum moldavica* (Khattulanuar et al. 2022). Recently, the cardioprotective effects of tilianin have considered much attention (Khattulanuar et al. 2022). Since ROS overproduction is associated with I/R damages. Tian *et al*. suggested that tilianin treatment promoted protective effects through activation of AMPK/SIRT1/PGC‐1α signaling pathway. Tilianin treatment enhanced PGC‐1α and modulated SOD enzyme showing antioxidant effects. Moreover, NRF‐1, TFAM, and forkhead transcription factor (FOXO1) expressions were upregulated (Tian et al. 2019) (Table 1) (Figure 1).

**TABLE 1:** Summary of the cardiovascular protective function of natural compounds through AMPK/SIRT1/PGC‐1α signaling pathway in in vivo studies.

Natural compound	Experimental model	Natural compound dose or concentration/route of exposure	Toxic compound dose or concentration/route of exposure	Finding(s)	Reference
In vivo studies					
Ferruginol	Doxorubicin cardiotoxicity model, C57BL/6 mice	20 mg/kg, oral, 35 days	Doxorubicin, 5 mg/kg, i.v., 4 days	Increased SIRT1 and PGC‐1α	Li, Cao, et al. (2021)
Tilianin	Ischemia/reperfusion model, Sprague Dawley rats	5 mg/kg, oral, 7 days	—	Increased AMPK, SIRT1, and PGC‐1α	Tian et al. (2019)
6‐Gingerol	Arsenic trioxide cardiotoxicity, Male KunMing mice	20 mg/kg, oral, 7 days	Arsenic, 5 mg/kg, i.p., 7 days	Increased AMPK, SIRT1, PGC‐1α, and Bcl‐2 Decreased Bax, and caspase‐3	Yarmohammadi et al. (2023)
Rosmarinic acid	Diabetic cardiomyopathy, C57BL/6 mice	100 mg/kg/day, oral, 4 weeks	Streptozotocin, 30 mg/kg, i.p., Single dose	Increased SIRT1 and PGC‐1α	Rijzewijk et al. (2009)
	Septic cardiomyopathy model, C57BL/6 mice	100 mg/kg/day, oral, 8 days	Lipopolysaccharide, 5 mg/kg, i.p., Single dose	Increased SIRT1 and PGC‐1α	Li, Feng, et al. (2021)
Resveratrol	Diabetic cardiomyopathy model, SPF male SD rats	50 mg/kg, oral, 16 weeks	Streptozotocin, 35 mg/kg, i.p., Single dose	Increased SIRT1, PGC‐1α, and NRF‐1	Diao et al. (2021)
	Female Wistar rats	5 mg/kg/day, i.p., 4 weeks	Streptozotocin, 40 mg/kg, i.p., Single dose	Increased SIRT1 and PGC‐1α	Peng et al. (2023)
Quercetin	Myocardial infarction model, Male Sprague–Dawley rats	25, 50, 100 mg/kg, oral, 7 days	—	Increased SIRT1 and PGC‐1α	Gormaz, Quintremil, and Rodrigo (2015)
Syringin	Myocardial infarction model, Male Sprague–Dawley rats	50 mg/kg, i.p., 7 days	—	Increased PGC‐1α, NRF‐2, and HO‐1	Han et al. (2022)
Melatonin	Diabetic cardiomyopathy model, C57BL/6 mice	10 mg/kg, i.p., 10 days	Streptozotocin, 50 mg/kg, i.p., 5 days	Increased SIRT1 and PGC‐1α Decreased Drp‐1	Fang et al. (2018)
	Myocardial ischemia/reperfusion injury in type 1 diabetic, Male Sprague–Dawley rats	10 mg/kg, i.p., 10 days	Streptozotocin, 50 mg/kg, i.p., 3 days	Increased SIRT1, SIRT3, and PGC‐1α	Yu et al. (2017)
	Acute myocardial infarction model, Female Wistar rats	5, 10, 20, and 40 mg/kg, i.p.	Isoproterenol, 25 mg/kg, s.c., Single dose	Increased SIRT1, SIRT3, PGC‐1α	Sun et al. (2023)
Puerarin	Cardiac hypertrophy model, Female Sprague–Dawley rats	50 mg/kg, 56 days	—	Increased PPARα, PGC‐1α, PGC1‐β, NRF‐1, and TFAM	Wang et al. (2019)
HuoXue QianYang QuTan Recipe	Male spontaneously hypertensive rats	38.7 g/kg, oral, 10 weeks	—	Increased PGC‐1α, SIRT1, NRF‐1, and TFAM	Mei et al. (2020)
Linggui Zhugan Decoction	Chronic heart failure model, SPF male SD rats	5.4 g/kg, oral, 6 weeks	—	Increased AMPK, SIRT1, PGC‐1α, NRF‐1, and TFAM	Hou et al. (2021)
Naringenin	Ischemia/reperfusion model, Sprague–Dawley rats	50 mg/kg, oral, 7 days	—	Increased AMPK, SIRT3, PGC‐1α, NRF‐1, TFAM, and Bcl‐2 Decreased Bax and caspase‐3	Yu et al. (2019)
Salidroside	Ischemia/reperfusion model, Sprague–Dawley rats	20, 40, mg/kg, oral, 5 days	—	Increased AMPK and PGC‐1α Decreased NF‐κB, PPAR‐α, TNF‐α, IL‐1β, and IL‐6	Ni et al. (2021)
	Diabetic cardiomyopathy model, C57BL/6 mice	50 and 100 mg/kg, oral, 64 days	Streptozotocin, 50 mg/kg, i.p., 5 days	Increased AMPK, SIRT3, PGC‐1α, NRF‐1, and TFAM	Diao et al. (2019)
Aconiti Lateralis Radix Praeparata	Heart failure model, Sprague–Dawley rats	9.0, 4.5, 0.9 g/kg/day, oral, 6 days	Propafenone, 10 mL/h	Increased NRF‐1, NRF‐2, and PGC‐1α	Yu et al. (2023)
Icariin	Ischemia/reperfusion model, Sprague–Dawley rats	60 mg/kg, oral, 14 days	—	Increased SIRT1 and Bcl‐2 Decreased Bax, caspase‐3, and FOXO1	Wu et al. (2018)
Benzoylaconine	Ischemia/reperfusion model, Sprague–Dawley rats	20 mg/kg, oral	—	Increased AMPK and PGC‐1α	Lu et al. (2017)
Danqi pill	Sprague–Dawley rats	0.75 g/kg/day, oral, 28 days	—	Increased NRF‐1, NRF‐2, PGC‐1α, and TFAM	Q. Zhang et al. (2020)
Songorine	Septic cardiomyopathy model, C57BL/6 mice	10, 50 mg/kg, i.p, 3 days	Lipopolysaccharide, 30 mg/kg, i.p., Single dose	Increased NRF‐1, HIF1 α, PGC‐1α, PPAR α, RXRα, and TFAM	Liu et al. (2017)
Tetrahydrocurcumin	Heart failure model, C57BL/6 mice	50 mg/kg, oral, 28 days	—	Increased PGC‐1α, and ATF5	Chen, Yan, and Zhang (2022)
Salvianolic acid B	Ischemia/reperfusion model, Sprague–Dawley rats	32 mg/kg, i.v.	—	Increased PGC‐1α, AMPK, and SIRT1	Li et al. (2022)

Bakuchiol is a monoterpene phenol and an analog of resveratrol found in the seeds of *Psoralea corylifolia* (Wang et al. 2018). Bakuchiol has many pharmacological effects, such as anti‐inflammatory, antioxidant, and antiapoptotic effects (Wang et al. 2018). Bakuchiol induced cardioprotective effects in an experimental I/R model through upregulating SIRT1 and PGC‐1α expressions. Moreover, bakuchiol treatment reduced apoptosis by inducing antiapoptotic factor Bcl‐2 and reduction in the level of pro‐apoptotic factor Bax, which presumably are attributed to SIRT1 induction effects of bakuchiol (Wang et al. 2018) (Table 2). Moreover, the results showed that bakuchiol treatment enhanced mitochondrial function by reducing mitochondrial oxidative injury through the SIRT1/PGC‐1α signaling pathway (Wang et al. 2018). FoxO proteins are expressed in various cell types, specifically in cardiomyocytes (Li et al. 2020). They play a crucial role in cellular functions, such as apoptosis, inflammation, and oxidative stress. Accordingly, a recent study revealed that icariin increased FOXO1 deacetylation by SIRT1, which increased MnSOD activity and reduced oxidative stress injuries. Moreover, it has been indicated that SIRT1/FOXO1 signaling pathway inhibits cell apoptosis (Wu et al. 2018). However, administration of siRNA and sirtinol reduced icariin effects on MnSOD activity. Hence, the expression of MnSOD may be controlled by the activity of FOXO1 (Wu et al. 2018) (Table 1) (Figure 1).

**TABLE 2:** Summary of the cardiovascular protective function of natural compounds through AMPK/SIRT1/PGC‐1α signaling pathway in in vitro studies.

Natural compound	Experimental model	Natural compound dose or concentration	Toxic compound dose or concentration/route of exposure	Finding(s)	Reference
In vitro studies					
Bakuchiol	Isolated rat heart	0.25, 0.5, and 1 μM, 5 min	—	Increased SIRT1 and PGC‐1α	Wang et al. (2018)
Isosteviol	H9C2 cells	5, 10, and 50 μM, 48 h	10 μM and 33.5 mM glucose for 48 h	Increased AMPK, SIRT1, and PGC‐1α	Naaz et al. (2020)
Rosmarinic acid	H9C2 cells	20 mM, 6 h	5.5 mM or 25 mM glucose for 48 h	Increased SIRT1 and PGC‐1α	Rijzewijk et al. (2009)
Naringenin	H9C2 cells	80 μM, 6 h	—	Increased AMPK, SIRT3, PGC‐1α, Bcl‐2 Decreased Bax, and caspase‐3	Yu et al. (2019)
Baicalin	Human fibroblast MRC‐5 cells	24 h	—	Increased VEGF, PGC‐1α, and ERRα	Arany et al. (2008)
Icariin	Primary neonatal rat ventricular myocytes	2, 4, and 8 μM, 24 h	—	Increased PGC‐1α, SIRT1, and Bcl‐2 Decreased Bax and caspase‐3	Wu et al. (2018)
Benzoylaconine	H9C2 cells	25, 50, and 75 μM, 6 h	—	Increased AMPK and PGC‐1α	Lu et al. (2017)
Sulforaphane	H9C2 cells	1, 5, and 10 μM, 24 h	—	Increased HO‐1, Bcl‐2 and PGC‐1α Decreased caspases 3/7	Fernandes et al. (2015)
Resveratrol	Primary neonatal rat cardiomyocytes	20 μM, 12 h	—	Increased PGC‐1α, SIRT3, SIRT1, and MFN1 Decreased DRP1	Tang et al. (2019)
Melatonin	H9C2 cells	0.01, 0.1, 1, and 10 mM	—	Increased PGC‐1α, NRF‐2, HO‐1, and Bcl‐2 Decreased caspase‐3, TNF‐α, IL‐6	Pourbarkhordar et al. (2024)
Acacetin	Primary neonatal rat cardiomyocytes	0.3, 1, and 3 μM	—	Increased NRF‐2, HO‐1, Bcl‐2, PGC‐1α, PPARα, and pAMPK Decreased Bax and IL‐6	Cui et al. (2022)

As a naturally occurring hormone found in plants and animals, melatonin has gained much attention for its positive effects on mitochondrial biogenesis (Baltatu et al. 2017; Pourbarkhordar et al. 2024; Rahmani, Roohbakhsh, Pourbarkhordar, Hayes, and Karimi 2024). In this accordance, a study showed that melatonin protected H9c2 cells against OGD/R‐induced oxidative damage, inflammation, and apoptosis through the PGC‐1α/NRF‐2 and PGC‐1α/TNF‐α signaling pathways (Zhi et al. 2020) (Table 1).

Salidroside, the main active component of *Rhodiola rosea*, has beneficial effects on CVDs (Ni et al. 2021). It was indicated that salidroside alleviated I/R injury by inflammation suppression through AMPK/PGC‐1α and AMPK/NF‐κB pathways (Chang et al. 2016) (Table 1). Quercetin, a well‐known naturally derived flavonol found in vegetables, fruits, and tea, has garnered significant interest recently for its cardioprotective effects, as evidenced by numerous studies (Mirsafaei et al. 2020; Zhou et al. 2022; Gormaz, Quintremil, and Rodrigo 2015). It was indicated that quercetin improved MI/R damages via activation of SIRT1/PGC‐1α signaling pathway (Tang et al. 2019). Moreover, it enhanced apoptotic‐related proteins by upregulating and downregulating Bcl‐2 and Bax, respectively (Tang et al. 2019) (Table 1).

Mitochondria are constantly changing cell structures that experience the process of fission (division of a mitochondrion) or fusion (joining two single mitochondrion). The equilibrium between fusion and fission processes plays a crucial role in dictating the quantity, morphology, and spatial arrangement of both individual mitochondria and interconnected mitochondrial networks (Rahmani, Roohbakhsh, and Karimi 2023). The activity of various GTPases controls fusion and fission. For example, MFN1, MFN2, and optic atrophy protein 1 (OPA1) play a key role during fusion, while Dynamin‐related protein 1 (DRP1) regulates the fission process. Indeed, mitochondria eliminate abnormal mitochondria through a process called mitophagy, which is regulated by PINK1 and Parkin proteins. Interestingly, it has been indicated that PGC‐1α enhances MFN2 levels and shifts mitochondrial dynamics toward fusion/mitophagy (Zheng et al. 2022). Accordingly, Zheng et al. (2022) revealed that resveratrol increased mitochondrial membrane potential (MMP) and ATP levels in cardiomyocyte mitochondria, which presumably contributes to the SIRT1/SIRT3‐FOXO pathway activation. Selisistat, a selective SIRT1 inhibitor, reversed resveratrol effects on sirtuins. Moreover, resveratrol promoted mitochondrial fusion by upregulation of MFN1, MFN2, and OPA1 in cardiomyocytes. Indeed, the results showed that resveratrol induced mitophagy through a Parkin‐dependent pathway (Table 2).

As part of the inflammatory immune response, the NOD‐like receptor protein 3 (NLRP3) inflammasome is closely related to CVDs. Numerous studies have confirmed that NLRP3 inflammasome is involved in the development of MI/R injury, cardiomyopathy, arrhythmia, and other CVDs. Mitochondrial injuries may trigger NLRP3 inflammasome activation. A recent study explored the effects of salvianolic acid B, an active component of *S. miltiorrhiza* on MI (Li et al. 2022). The results indicated that salvianolic acid B reduced the NLRP3 inflammasome during MI through AMPK/SIRT1/PGC‐1α signaling pathway regulation (Li et al. 2022). EX527, a selective SIRT1 inhibitor, prevented the NLRP3 inflammasome inactivation by salvianolic acid B and reversed its anti‐inflammatory effects (Li et al. 2022) (Table 1) (Figure 1).

### 2.2 Septic Cardiomyopathy

Sepsis is a life‐threatening dysregulated systemic inflammation caused by infection, which accounts for 200,000 deaths per year in the United States (Liu et al. 2017). Sepsis‐induced cardiomyopathy is one of the serious complications of severe sepsis, characterized by mitochondrial dysregulation and heart contraction impairment (Li, Feng, et al. 2021). Songorine is an alkaloid derived from *Aconitum carmichaelii*, with significant anti‐inflammatory and cardioprotective properties (Li, Feng, et al. 2021). Li *et al*. showed that songorine reduced oxidative stress induced by lipopolysaccharides (LPS) through enhanced Keap1 degradation to preserve NRF‐2 stability. Moreover, the results indicated that songorine promoted fatty acid β‐oxidation and tricarboxylic acid (TCA) cycle. Songorine treatment also enhanced TFAM through NRF‐1 activation. This effect was abrogated by NRF‐1 knockdown. As a key finding, NRF‐2 transcriptionally regulates mitochondrial genes in cooperation with PGC‐1α (Li, Feng, et al. 2021). Peng et al. (2023) showed that rosmarinic acid improved mitochondrial injuries induced by LPS in cardiomyocytes, as indicated by increasing MMP and ATP levels, and mitochondrial ultrastructure improvement. Such effects were associated with the SIRT1/PGC‐1α pathway activation as SIRT1 inhibition reversed rosmarinic acid effects (Table 1) (Figure 1).

### 2.3 Diabetic Cardiomyopathy

Diabetes mellitus is a long‐term metabolic disorder that is becoming more common and may result in various pathological complications (Akgun‐Unal et al. 2023). Diabetes mellitus affects 380 million people worldwide and is expected to rise to 439 million by 2030 (Akgun‐Unal et al. 2023). Importantly, diabetes mellitus is associated with an increased risk of CVD including coronary artery disease, congestive heart failure, acute MI, and cardiomyopathy (Yu et al. 2017). Diabetic cardiomyopathy is a serious complication of diabetes mellitus that leads to heart failure in severe cases. Initially, it causes diastolic dysfunction, but as it progresses, it may result in systolic dysfunction. Recent research has highlighted the importance of mitochondrial dysfunction and excessive ROS production in the development of diabetic cardiomyopathy (Diao et al. 2019). A study showed that salidroside could increase MnSOD activity by enhancing SIRT3 expression. Indeed, it was indicated that salidroside promoted SIRT3 translocation from the cytoplasm to mitochondria. A SIRT3 siRNA reversed the protective effects of salidroside on mitochondrial oxidative stress. Moreover, the results showed that salidroside increased AMPK phosphorylation, which enhanced PGC‐1α/TFAM expression. Salidroside also improved systolic dysfunction and elevated brain natriuretic peptide (BPN) levels (Li, Wei, et al. 2021).

During diabetic cardiomyopathy, the metabolic pathway shifts from glucose oxidation to fatty acid β oxidation. This shift increases ROS production, which stimulates apoptosis (Rijzewijk et al. 2009). Supporting this notion, Diao et al. (2021) showed that rosmarinic acid, a water‐soluble polyphenolic acid extracted from rosemary, improved glucose oxidation, which was associated with SIRT1 activation. Moreover, rosmarinic acid diminished ROS generation and apoptosis caused by diabetic cardiomyopathy, while upregulating SIRT1 and PGC‐1α expressions. Indeed, SIRT1 suppression by Ad‐sh‐SIRT1 neutralized rosmarinic acid effects (Diao et al. 2021). Therefore, it was suggested that rosmarinic acid cardioprotective effects were mediated through SIRT1/PGC‐1α pathway activation (Diao et al. 2021) (Table 1). Another study showed that resveratrol treatment ameliorated cardiac injuries by regulating the SIRT1/PGC‐1α pathway and oxidative stress reduction in an experimental diabetic cardiomyopathy model. The results showed that resveratrol regulated PGC‐1α deacetylation through SIRT1 (Fang et al. 2018). Both pharmacological inhibition of SIRT1 and siRNA transfection against SIRT1 reversed resveratrol effects (Fang et al. 2018). Moreover, resveratrol treatment showed beneficial effects on insulin sensitivity indicated by fasting blood glucose and hemoglobin A1c (HbA1c) reductions (Fang et al. 2018). Interestingly, the cardioprotective effects of resveratrol were associated with mitochondrial function improvement indicated by improvement in ATP generation, lower mtDNA content, and upregulation of PGC‐1α and NRF expressions (Fang et al. 2018).

Recent studies show that in response to CVD injuries and increased oxidative stress and apoptosis levels, mitochondria undergo mitochondrial fission (Rahmani, Roohbakhsh, and Karimi 2023). Ding et al. (2018) showed that melatonin administration reduced mitochondrial fission mediated by DRP1. This effect of melatonin was mediated via the activation of the SIRT1/PGC‐1α signaling pathway. Furthermore, DRP1 inhibition suppressed mitochondrial oxidative stress. Similarly, Akgun‐Unal et al. (2023) showed that the concomitant use of melatonin and resveratrol improved oxidative stress in a diabetic cardiomyopathy model. The results suggested SIRT1 activation contributed to this effect. However, coadministration of the compounds did not produce a greater effect than independent administration (Table 1) (Figure 1).

### 2.4 Myocardial Infarction

Myocardial infarction is a major contributor to global mortality. According to the latest reports, it accounts for one‐seventh of all deaths (Sun et al. 2023). The pathological mechanism(s) of myocardial infarction remains unclear, but recent studies highlight the role of ROS in the progression of myocardial infarction (Sun et al. 2023). A study showed that melatonin treatment reduced mitochondrial damage through regulation of both SIRT1 and SIRT3. SIRT3 activation decreased oxidative stress by enhancing MnSOD activity and GSH levels. Furthermore, upregulation of PGC‐1α enhanced the mitochondrial biogenesis (Naaz et al. 2020) (Figure 1).

### 2.5 Heart Failure

A leading cause of death worldwide is heart failure following cardiac hypertrophy (Mei et al. 2020). Cardiac hypertrophy is a condition in which the heart compensates for an increased workload. Various heart diseases and conditions can cause it. Physiological hypertrophy happens as a natural response to physiological stimuli, while pathological hypertrophy occurs due to hemodynamic stress such as hypertension or myocardial infarction. Pathological hypertrophy is often associated with fibrosis and may lead to cardiac dysfunction (Mei et al. 2020). Among subcellular organelles, mitochondria are key targets of ROS attack associated with cardiac hypertrophy (Wang et al. 2019). Impairment of mitochondrial biogenesis is an important phenomenon of cardiac hypertrophy (Wang et al. 2019). Considering the influence of mitochondrial biogenesis during cardiac hypertrophy and the profound impact of SIRT1/PGC‐1α signaling on mitochondrial biogenesis and metabolism, Mei et al. (2020) showed that isosteviol (a terpenoid derived from stevioside) exerted a cardioprotective effect in isoprenaline/high glucose (ISO‐/HG)‐induced myocardial hypertrophy. Isosteviol induced such protective effects by reducing oxidative stress and ROS‐induced mitochondrial fission via activation of SIRT1/PGC‐1α signaling pathway (Table 2). Another study indicated that PGC‐1α influenced the expression of genes related to oxidative phosphorylation, ATP synthesis, and fatty acid oxidation involved in cell metabolism. Hou et al. (2021) demonstrated that puerarin, a phytoestrogen and the active ingredient of *Pueraria radix*, reduced cardiac hypertrophy through enhancing the expression of PPARα, PGC‐1α, and PGC‐1β. Moreover, they showed that puerarin increased fatty acid β‐oxidation gene expressions such as carnitine palmitoyl transferase 1A, carnitine palmitoyl transferase 1B, and long‐chain acyl‐CoA dehydrogenase (Hou et al. 2021). Additionally, puerarin treatment increased the expression of estrogen‐related receptor (ERR)α, NRF‐1, and TFAM proteins associated with mitochondrial biogenesis (Hou et al. 2021) (Figure 1).

A recent study on a rat model of chronic heart failure showed that treatment with Linggui Zhugan decoction upregulated NRF‐1 and TFAM levels, two essential proteins downstream of PGC‐1α (Yu et al. 2023). Moreover, the phosphorylated form of AMPK and the expression levels of SIRT1 and PGC‐1α were significantly increased. This showed that Linggui Zhugan decoction activated the SIRT1/AMPK/PGC‐1α pathway to protect mitochondria and resist oxidative stress in myocardial cells (Yu et al. 2023). Interestingly, captopril, a widely recognized angiotensin‐converting enzyme inhibitor, was compared to Linggui Zhugan decoction. The repair of mitochondrial damage in myocardial cells of rats with heart failure was observed after administering captopril. However, there was no impact on the protein and mRNA levels of SIRT1, p‐AMPK, and PGC‐1α (Yu et al. 2023). Another study investigated the cardioprotective effects of two herbal Chinese *Aconiti Lateralis Radix Praeparata* and *Zingiberis Rhizoma* on a heart failure rat model induced by propafenone hydrochloride. The combination of these substances has been shown to reduce the cardiovascular biomarkers such as LDH, BNP, and cardiac troponin T. Likewise, the results indicated that treatment with these herbs increased the levels of NRF‐1 and NRF‐2. The study indicated that this coadministration had a greater impact on NRF‐1 than on NRF‐2. Moreover, these herbs increased mitochondrial biogenesis through upregulation of SIRT1 and PGC‐1α (Lu et al. 2017) (Table 1). Chen, Yan, and Zhang (2022) found that benzoylaconine, an alkaloid bioactive compound in Fuzi could alleviate cardiomyocyte injury by AMPK/PGC‐1α activation (Table 1).

Mitochondrial unfolding protein responses (UPR) are mainly activated under pathological conditions when mitochondrial protein disruption occurs. Consequently, the expression of proteins involved in mitochondria protection like HSP10, HSP60, and proteases improves. In accordance, Zhang et al. (2020) showed that tetrahydrocurcumin, a main metabolite of curcumin, improved cardiac function and reduced oxidative stress in a cardiac hypertrophy model via PGC‐1α upregulation. Furthermore, tetrahydrocurcumin enhanced ATF5 and several UPR effectors (Table 1) (Figure 1).

## 3 The Protective Effects of Natural Compounds Through AMPK/SIRT1/PGC‐1α Pathway in Vascular Diseases

### 3.1 Angiogenesis

The process of making new blood vessels is called angiogenesis. Aberrant angiogenesis may lead to pathological conditions such as I/R, hypertension, and neurodegeneration. Additionally, excessive angiogenesis has been linked to cancer, psoriasis, arthritis, and blindness. Vascular endothelial growth factor (VEGF) is a crucial element in angiogenesis, as its elevation promotes the formation of new blood vessels (Arany et al. 2008).

ERRs are typically viewed as constitutively active receptors that interact with coactivators even without exogenous ligands (Zhang et al. 2011). PGC‐1α interacts with ERRα to induce VEGF expression and angiogenesis through a hypoxia‐inducible factor‐1 alpha (HIF‐1α)‐independent mechanism (Zhang et al. 2011). Another study by Zhang et al. (2011) showed that baicalin, a major component of *Scutellaria baicalensis* root, increased VEGF expression through the activation of ERRα/PGC‐1α pathway (Figure 1).

## 4 The Protective Effects of Natural Compounds Through AMPK/SIRT1/PGC‐1α Pathway in Cardiovascular Toxicities

### 4.1 Doxorubicin Cardiotoxicity

Doxorubicin (Dox), also known as adriamycin, is a widely used chemotherapy drug for treating various cancers such as breast, ovary, bladder, and thyroid. However, its clinical use is limited due to its significant cardiotoxicity (Eisvand et al. 2022). Mitochondrial dysfunction is a crucial factor in the development of cardiotoxicity caused by Dox. Thus, protecting mitochondria may be a potential solution. Currently, dexrazoxane is the only available treatment for Dox‐induced cardiotoxicity. However, its use may lead to myelosuppression or secondary malignancies. Therefore, exploring new natural compounds with fewer side effects is important to protect the heart against Dox toxicity.

Ferruginol is a natural polyphenol and terpenoid derived from *S. sempervirens* that has cardioprotective effects (Zhang et al. 2021). Li, Cao, et al. (2021) showed that ferruginol treatment reduced cardiotoxicity induced by Dox through enhancing the SIRT1/PGC‐1α pathway leading to mitochondrial biogenesis and fatty acid oxidation. The results indicated that ferruginol only enhances the expression of PGC‐1α without improving the expression of PPARα. Using selisistat and SR‐18292 as inhibitors of SIRT1 and PGC‐1α, they confirmed the effects of ferruginol on mitochondrial biogenesis and fatty acid oxidation through SIRT1/PGC‐1α pathway (Table 1) (Figure 1).

### 4.2 Arsenic Cardiotoxicity

Arsenic, a highly toxic compound called the “king of poisons”, is found in foods and the environment (Yarmohammadi et al. 2023). Chronic exposure to arsenic causes CVDs such as ischemia, arrhythmia, and heart failure (Yarmohammadi et al. 2023). Ginger, a widely used condiment, has many pharmacological effects like antioxidant, anti‐inflammatory, and antibacterial effects. One of the important aromatic constituents of ginger is 6‐gingerol, a naturally occurring phenol that exhibits several health benefits. Han et al. (2022) explored the cardioprotective effects of 6‐gingerol in a mouse model of arsenic‐induced cardiotoxicity. The study indicated that 6‐gingerol exerted its cardioprotective effects through amelioration of oxidative stress by triggering SOD activity and suppression of ROS and MDA production. These effects were associated with AMPK/SIRT1/PGC‐1α pathway activation. Moreover, 6‐gingerol treatment reduced apoptosis by downregulation of caspase‐3 and upregulation of Bcl‐2 in arsenic‐treated mice (Table 1) (Figure 1).

## 5 Discussion

The natural compounds are widely recognized for their antioxidant properties. Their ability to act as antioxidants stems from their electron‐rich chemical structure or their direct impact on antioxidant enzymes like GSH, CAT, and SOD. Despite their significant therapeutic properties, natural compounds have low and infrequent toxicity profiles. A number of studies have shown that natural compounds can enhance antioxidant enzyme activity via mitochondria‐related pathways, including SIRT1/PGC‐1α/NRF pathway. In support of this, studies performed in various experimental models indicated that activation of these pathways induces several antioxidant enzymes such as MnSOD, GPX, and CAT. Mitochondria are considered the main source of ROS production, making protecting mitochondrial function an important target for compounds that reduce ROS production in mitochondria. In our literature review, we found that natural compounds in pathological conditions such as cardiomyopathy (Li, Feng, et al. 2021), I/R (Yu et al. 2019), and cardiac hypertrophy (Hou et al. 2021) could induce NRF‐1 and NRF‐2 which activate antioxidant‐responsive element (ARE). PGC‐1α signaling pathway not only regulates mitochondrial biogenesis, it also influences mitochondrial dynamic nature. Several mitochondrial proteins, including MFN‐1, MFN‐2, and DRP1, have been associated with this effect (Zheng et al. 2022; Ding et al. 2018). In the cardiovascular system, the primary source of ATP synthesis is fatty acid oxidation. As a result, any reduction in fatty acid metabolism leads to myocardial insufficiency. We found that natural compounds could enhance fatty acid oxidation, which is correlated with their effects on PGC‐1α, SIRT1, and NRF‐2 (Li, Feng, et al. 2021; Diao et al. 2021; Hou et al. 2021). Moreover, several studies found that activation of SIRT1/PGC‐1α signaling pathway contributes to the antiapoptotic effects of natural compounds (Wang et al. 2018; Tang et al. 2019; Han et al. 2022). Consequently, increasing antioxidant activity and antiapoptotic effects of natural compounds were correlated with positive effects on mitochondrial dynamics and biogenesis via PGC‐1α activation. It is also pertinent to mention that natural compounds also regulate upstream or downstream of the PGC‐1α, including SIRT1 and AMPK. This finding provides novel insight into the exact mechanism of natural compounds on the posttranslational modification of PGC‐1α. The induction of PGC‐1α expression is one of the key mechanisms by which natural compounds, such as rosmarinic acid, resveratrol, and syringin improve cardiovascular health. However, the mechanism(s) of action responsible for the observed improvements have not been studied in detail. Future research in this area will require a thorough investigation of the mechanisms that control the regulation of different cardiovascular‐specific forms of AMPK, SIRT1, and PGC‐1α, as well as their specific functions within particular regions of the heart.

However, despite the promising results obtained in preclinical studies, the results have not been explored in well‐controlled clinical trials. Therefore, translating experimental studies into clinical practice requires considerable effort. Finally, the present review indicates that activation of the AMPK/SIRT1/PGC‐1α pathway by selected natural compounds promotes heart health.

## 6 Conclusion and Prospective

This review focuses on the positive role of naturally occurring substances in enhancing mitochondrial function, particularly by influencing AMPK/SIRT1/PGC‐1α, which improves pathological states in different cardiovascular disease models. Natural compounds discussed in this article through enhancing AMPK/SIRT1/PGC‐1α signaling pathway and downstream proteins such as NRF‐1, NRF‐2, ERRa, and TFAM promote oxidative phosphorylation, antioxidant, antiapoptotic, and anti‐inflammatory effects. Given the key role of PGC‐1 coactivators in CVDs, the AMPK/SIRT1/PGC‐1α represents a novel potential therapeutic target for CVD treatment by natural compounds. Furthermore, natural compounds have a long history of being utilized in clinical settings without apparent safety issues. Consequently, they may present an optimal alternative in the future for the prevention and treatment of mitochondrial‐related cardiovascular diseases.

## Author Contributions

**Sohrab Rahmani:** writing – original draft (equal), writing – review and editing (equal). **Ali Roohbakhsh:** writing – review and editing (equal). **Vahid Pourbarkhordar:** writing – original draft (equal). **Gholamreza Karimi:** conceptualization (equal).

## Conflicts of Interest

The authors declare no conflicts of interest.

## Data Availability

Data are available as request.
