# EXPRESSION OF CONCERN: The Cardiovascular Protective Function of Natural Compounds Through AMPK/SIRT1/PGC‐1α Signaling Pathway

**DOI:** 10.1002/fsn3.71643

**Published:** 2026-03-18

**EXPRESSION OF CONCERN**: S. Rahmani , A. Roohbakhsh , V. Pourbarkhordar , and G. Karimi , “The Cardiovascular Protective Function of Natural Compounds Through AMPK/SIRT1/PGC‐1α Signaling Pathway,” Food Science & Nutrition 12, no. 12 (2024): 9998–10,009, 10.1002/fsn3.4553.PMC1166681539723061

This Expression of Concern is for the above article, published online on 08 November 2024 in Wiley Online Library (wileyonlinelibrary.com), and has been issued by agreement between the journal Editor‐in‐Chief, Y. Martin Lo; and Wiley Periodicals LLC. The Expression of Concern has been agreed due to concerns raised by third parties. The reference Tang et al. (2019; https://doi.org/10.1002/jcb.28255) had been retracted prior to the publication of this article, and any statements in the article relying on that work should therefore be disregarded. In addition, Tables 1 and 2 contain several inconsistencies between the listed entries and their corresponding cited references, and the introduction includes citations to the authors' own previous work that are only marginally relevant to the context. As these issues do not affect the overall conclusions of the study, the journal has decided to issue an Expression of Concern to inform and alert the readers.

